# A Review of Sponge-Derived Diterpenes: 2009–2022

**DOI:** 10.3390/md22100447

**Published:** 2024-09-28

**Authors:** Jinmei Xia, Xiangwei Chen, Guangyu Li, Peng Qiu, Weiyi Wang, Zongze Shao

**Affiliations:** 1 Key Laboratory of Marine Biogenetic Resources, Third Institute of Oceanography, Ministry of Natural Resources, Xiamen 361005, China; cxw18737378901@163.com (X.C.); liguangyu@tio.org.cn (G.L.); f1932193525@126.com (P.Q.); 2 Department of Pharmacy, NO. 971 Hospital of the People’s Liberation Army Navy, Qingdao 266000, China

**Keywords:** sponge, marine, diterpene, biological activity

## Abstract

Sponges are a vital source of pharmaceutically active secondary metabolites, of which the main structural types are alkaloids and terpenoids. Many of these compounds exhibit biological activities. Focusing specifically on diterpenoids, this article reviews the structures and biological activities of 228 diterpenes isolated from more than 33 genera of sponges from 2009 to 2022. The *Spongia* sponges produce the most diterpenoid molecules among all genera, accounting for 27%. Of the 228 molecules, 110 exhibit cytotoxic, antibacterial, antifungal, antiparasitic, anti-inflammatory, and antifouling activities, among others. The most prevalent activity is cytotoxicity, present in 54 molecules, which represent 24% of the diterpenes reported. These structurally and biologically diverse diterpenoids highlight the vast, yet largely untapped, potential of marine sponges in the discovery of new bioactive molecules for medicinal use.

## 1. Introduction

Sponges are important multicellular animals, exhibiting a vast diversity and widespread distribution. More than a decade ago, there were already about 15,000 species of sponges that had been described [1]. The number of natural products derived from sponges is substantial, with over 200 new molecules discovered each year [2,3,4]. Compounds derived from sponges exhibit a rich diversity, encompassing terpenoids [5], sterols, alkaloids [6], ceramides, macrolides, peptides, and others. Among these, alkaloids and terpenoids are the most abundant, accounting for 50% of the total [7]. Diterpenoids represent a class of structurally diverse terpenes with various biological activities.

There have been some reviews on the separation, structural elucidation, and biological activity evaluation of diterpenoids. From 1984 to 2009, Hanson James R. published a series of reviews on newly discovered diterpenes every year [8,9,10,11]. Since 2011, Hanson began publishing reviews on diterpenoids from terrestrial sources [12]. Reviews on diterpenoids from marine sources are relatively fewer.

We have consistently focused on the diterpene compounds derived from marine sources. In our review of marine-derived diterpenes, we found that fungi and sponges are primary sources of these compounds. Previously, we summarized the diterpenes from marine fungi [13]. Here, we provide another review focusing on the diterpenes from marine sponges.

There are some review articles on sponge-derived natural products. Two articles summarize the chemical and biological diversity of compounds from sponges over a decade, with the timespans being 2009–2018 [7] and 2011–2020 [14], respectively. Some reviews focus on specific types of sponges, such as molecules from the order Dictyoceratida [15], the families Hymedesmiidae [16] and Latrunculiidae [17], and the genera *Amphimedon* [18], *Aaptos* [19], *Callyspongia* [20], *Haliclona* [21], *Petrosia* [22], *Phorbas* [23], *Reniera* [24], *Stelletta* [25], and *Suberea* [26]. Other reviews focus on compounds with specific activities, like sponge-derived molecules with antimalarial/antiprotozoal activity [27] and natural products from Red Sea sponges with cytotoxic activity [28]. A review critically analyzed drugs/drug candidates inspired by natural sponge products [29]. There are also reviews focusing on specific types of compounds, such as alkaloids from sponges [6]. Diterpenes sourced from sponges have not been reviewed yet.

In this article, the structures and bioactivities of 228 newly discovered diterpenoids from more than 33 genera of sponges reported by 73 publications covering 14 years, from 2009 to 2022, are summarized.

## 2. The Characteristics of Diterpenes from Sponges

**Figure 1:**
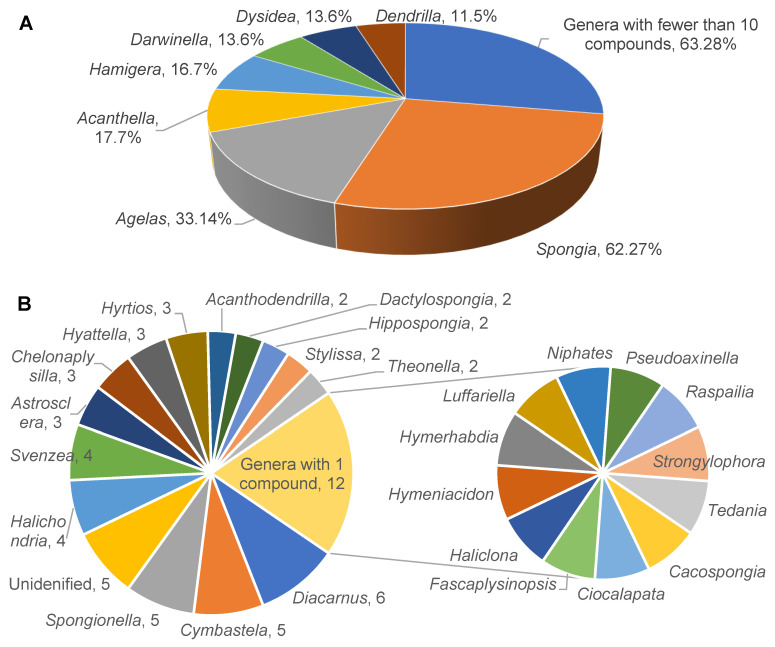
Number of new diterpenoids isolated from each genus of sponges from 2009 to 2022. (**A**) Overview of diterpenoid distribution across all genera, with those having fewer than 10 compounds grouped. (**B**) Close-up of genera with fewer than 10 diterpenoids each.

From 2009 to 2022, there were 73 reports on 228 new diterpenoids from sponges. Among these compounds, 62 were from the genus *Spongia*, accounting for 27% of the total, while 33, 17, 16, 13, 13, and 11 were from the genera *Agelas*, *Acanthella*, *Hamigera*, *Darwinella*, *Dysidea*, and *Dendrilla*, respectively (Figure 1A). In addition, 26 other genera of sponges have also yielded diterpenoids, with each contributing fewer than 10 molecules (Figure 1B). Among these, 12 genera have each produced a single diterpenoid. Furthermore, there are five diterpenoids that originate from sponges that have not yet been identified at the genus level.

Among these diterpenes, 110 have demonstrated a variety of bioactivities, constituting 48% of the overall number of reported compounds. The literature has reported 138 bioactivity data entries for these compounds, with 17, 4, and 1 compounds exhibiting two, three, and four different types of bioactivities, respectively. Cytotoxic activities against various tumor cell lines are considered to be a single type of activity.

**Figure 2:**
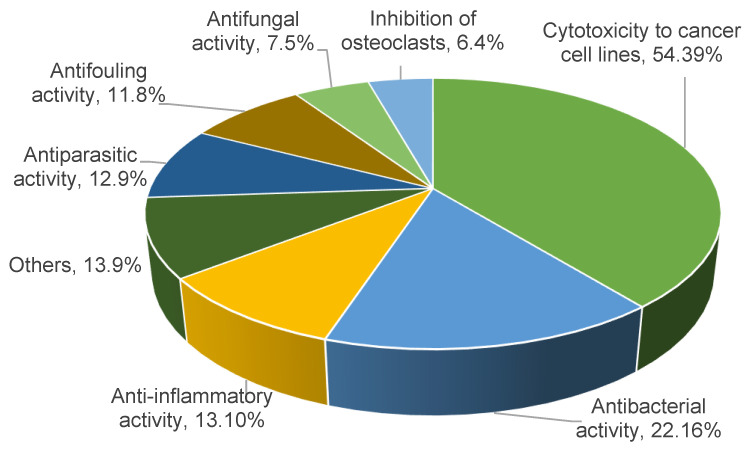
Bioactivity distribution of sponge-derived diterpenoids from 2009 to 2022.

The most frequently reported activity was cytotoxicity against tumor cells, with 54 molecules showing this activity, accounting for 39% of the activity entries and 24% of all reported molecules (Figure 2). The number of compounds with antibacterial activity ranked second, with 22 molecules, among which 6 had antituberculosis activity. The numbers of molecules with anti-inflammatory, antiparasitic, antifouling, antifungal, and osteoclast-inhibitory activities were 13, 12, 11, 7, and 6, respectively. Among the 12 molecules with antiparasitic activity, 9 were active against *Plasmodium* species, whereas 3 were active against the leishmaniasis parasite. The activities of 13 molecules were categorized as other types, including radiation sensitization activity, antioxidant activity, inhibitory activity against specific target proteins such as Casitas B-lineage lymphoma proto-oncogene-b (Cbl-b), antiviral activity, and so on.

## 3. Isolation, Structure, and Bioactivities of Diterpenes from Sponges

### 3.1. Acanthella

**Figure 3:**
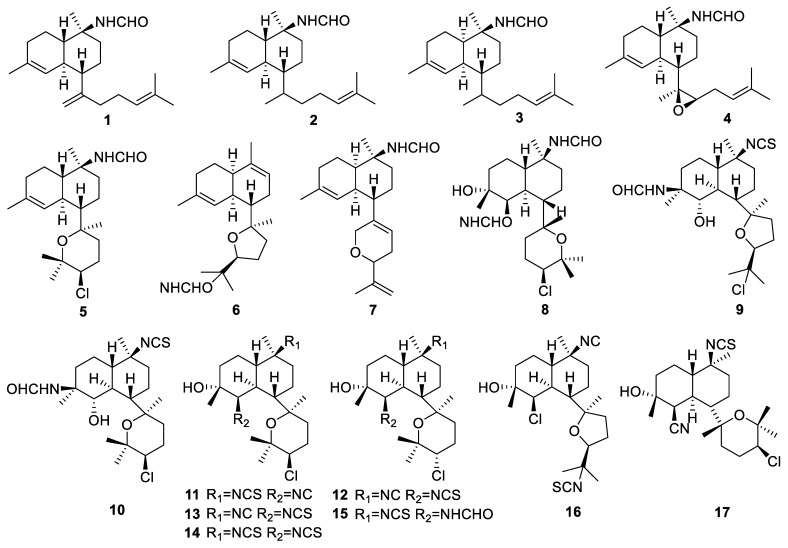
Chemical structures of diterpenes from *Acanthella* sponges (**1**–**17**).

A total of 17 diterpenes were obtained from *Acanthella* sponges. Seven formamido-diterpenes—cavernenes A–D, kalihinenes E–F, and kalihipyran C (**1**–**7**, Figure 3)—were isolated from the South China Sea sponge *Acanthella cavernosa* [30]. The cytotoxicity of these compounds was evaluated using five human cancer cell lines (human colon cancer cell line HCT-116, human lung epithelial cell line A549, human cervical carcinoma cell line HeLa, human hepatocellular carcinoma cell line QGY-7701, and human mammary cancer cell line MDA-MB-231).

Compounds **1** and **2** showed cytotoxic activities against HCT-116 cells, with IC_50_ (50% inhibitory concentration) values of 6.31 and 8.99 µM, respectively (Table 1). Compound **5** showed cytotoxic activity against HCT-116, HeLa, QGY-7701, and MDA-MB-231 cells, with IC_50_ values of 14.36, 13.36, 17.78, and 12.84 µM, respectively.

A nitrogen-containing kalihinane-type diterpenoid, bisformamidokalihinol A (**8**, Figure 3), was obtained from the *Acanthella cavernosa* sponge [31]. Eight diterpenoids, kalihinols M–T (**9**–**16**, Figure 3), were isolated from the South China Sea sponge *Acanthella cavernosa* [32]. Kalihinols M–N, with a formamide functionality at C-4, extended the structural breadth of this diterpenoid family. Kalihinols O–R showed cytotoxic activity against HCT-116 cells, with IC_50_ values of 5.97, 10.68, 20.55, and 13.44 µM, respectively. Kalihinol P displayed cytotoxic activity against H1299 cells, with an IC_50_ value of 26.21 µM. Kalihinols O–T displayed significant antifouling activity against *Balanus amphitrite* larvae, with EC_50_ values of 1.43, 0.72, 1.48, 1.16, 0.53, and 0.74 µM, respectively. A kalihinol diterpene, 10-epi-kalihinol X (**17**, Figure 3), was obtained from the Hainan sponge *Acanthella* sp. [33]. Compound **17** exhibited in vitro cytotoxicity against the human lung adenocarcinoma cell line A549, with an IC_50_ value of 9.30 µg/mL.

**Table 1:** Sponge-derived diterpenes with various bioactivities.

Compound Number	Compound Name	Producing Sponge	Activity	Reference
			Cytotoxicity to cancer cell lines	
**1**–**2**	Cavernenes A–B	*Acanthella cavernosa*	Cytotoxicity against the HCT-116 cell line, with IC_50_ values of 6.31 and 8.99 µM, respectively	[30]
**5**	Kalihinene E	*Acanthella cavernosa*	Cytotoxicity against the HCT-116, HeLa, QGY-7701, and MDA-MB-231 cell lines, with IC_50_ values of 14.36, 13.36, 17.78, and 12.84 µM, respectively	[30]
**11**	Kalihinol O	*Acanthella cavernosa*	Cytotoxicity against HCT-116 cells, with an IC_50_ value of 5.97 µM	[32]
**12**	Kalihinol P	*Acanthella cavernosa*	Cytotoxicity against HCT-116 and H1299 cells, with IC_50_ values of 10.68 and 26.21 µM, respectively	[32]
**13**–**14**	Kalihinols Q–R	*Acanthella cavernosa*	Cytotoxicity against HCT-116 cells, with IC_50_ values of 20.55 and 13.44 µM, respectively	[32]
**17**	10-*Epi*-kalihinol X	*Acanthella* sp.	Cytotoxicity against the A549 cell line, with an IC_50_ value of 9.30 µg/mL	[33]
**20**	Axistatin 1	*Agelas axifera* Hentschel	Cytotoxicity against P388, BXPC-3, MCF-7, SF-268, NCI-H460, KM20L2, and DU-145 cells, with GI_50_ (50% growth inhibition) values of 19.8, 4.8, 5.7, 3.6, 4.6, 4.1, and 4.8 µM, respectively	[34]
**21**	Axistatin 2	*Agelas axifera* Hentschel	Cytotoxicity against P388, BXPC-3, MCF-7, SF-268, NCI-H460, KM20L2, and DU-145 cells, with GI_50_ values of 22.8, 5.5, 6.8, 3.9, 4.3, 4.1, and 5.0 µM, respectively	[34]
**22**	Axistatin 3	*Agelas axifera* Hentschel	Cytotoxicity against P388, BXPC-3, MCF-7, SF-268, NCI-H460, KM20L2, and DU-145 cells, with GI_50_ values of 8.9, 6.0, 5.8, 3.5, 5.4, 6.9, and 7.5 µM, respectively	[34]
**27**	Nemoechine G	*Agelas* aff. *nemoechinata*	Cytotoxicity against Jurkat cell lines, with an IC_50_ of 17.1 µM	[35]
**28**	Nemoechine D	*Agelas* aff. *nemoechinata*	Cytotoxicity against the human promyelocytic leukemia HL-60 cell line, with an IC_50_ value of 9.9 µM	[36]
**36**	(+)-Agelasine B	*Agelas mauritiana*	Cytotoxicity toward the cancer cell lines PC9, A549, HepG2, MCF-7, and U937, with IC_50_ values of 5.08, 14.07, 9.76, 7.64, and 4.49 µM, respectively	[37]
**40**	Iso-agelasine C	*Agelas nakamurai*	Cytotoxicity against HL-60, K562, and HCT-116 cell lines, with IC_50_ values of 25.3, 28.9, and 38.8 µM, respectively	[38]
**41**	Iso-agelasidine B	*Agelas nakamurai*	Cytotoxicity against HL-60 and K562 cell lines, with IC_50_ values of 33.0 and 39.2 µM, respectively	[38]
**42**	(−)-Agelasine D	*Agelas nakamurai*	Cytotoxicity against L5178Y mouse lymphoma cells, with an IC_50_ value of 4.03 µM	[39]
**43**	(−)-Agelamide D	*Agelas nakamurai*	Cytotoxicity against L5178Y mouse lymphoma cells, with an IC_50_ value of 12.5 µM	[39,40,41]
**42**	(−)-Agelasine D	*Agelas nakamurai*	Cytotoxic to Hep3B cells, with a GI_50_ of 9.9 µM	[41]
**43**	(−)-Agelamide D	*Agelas nakamurai*	Cytotoxic to Hep3B cells, with a GI_50_ of 12.0 µM	[41]
**99**	NN *	*Dysidea* cf. *arenaria*	Cytotoxicity against NBT-T2 rat bladder epithelial cells, with an IC_50_ value of 1.9 µg/mL	[42]
**103**–**104**	NN	*Dysidea* cf. *arenaria*	Cytotoxicity against NBT-T2 rat bladder epithelial cells, with IC_50_ values of 1.8 and 4.2 µg/mL, respectively	[42]
**105**–**108**	NN	*Dysidea* cf. *arenaria*	Cytotoxicity against the NBT-T2 cell line, with IC_50_ values of 3.1, 1.9, 8.4, and 3.1 µM, respectively	[43]
**112**	2,5-Dihydroxy-homoverrucos-(3)-ene	*Halichondria* sp.	Cytotoxicity against the human multiple myeloma cell line RPMI-8266, with an IC_50_ of 49.0 µM	[44]
**113**	2-Hydroxy-5-oxo-homoverrucos-(3)-ene	*Halichondria* sp.	Cytotoxicity against RPMI-8266 cells, with an IC_50_ of 65.8 µM	[44]
**114**	5,18-Dihydroxy-homoverrucosane	*Halichondria* sp.	Cytotoxicity against RPMI-8266 cells, with an IC_50_ of 32.7 µM	[44]
**115**	5-Hydroxy-18-aldehyde-homoverrucosane	*Halichondria* sp.	Cytotoxicity against RPMI-8266 cells, with an IC_50_ of 49.3 µM	[44]
**1** **16**	Halioxepine	*Haliclona* sp.	Cytotoxicity against NBT-T2 cells, with an IC_50_ of 4.8 µg/mL	[45]
**124**–**125**, **127**–**128**,**130**	Hamigerans M–Q	*Hamigera tarangaensis*	Cytotoxicity against the HL-60 cell line, with IC_50_ values of 6.9, 19.5, 14.7, 21.3, and 33.3 µM, respectively	[46]
**126**	18-*Epi*-hamigeran N	*Hamigera tarangaensis*	Cytotoxicity against the HL-60 cell line, with an IC_50_ value of 14.1 µM	[46]
**129**	18-*Epi*-hamigeran P	*Hamigera tarangaensis*	Cytotoxicity against the HL-60 cell line, with an IC_50_ value of 11.6 µM	[46]
**136**	3*β*-Hydroxyspongia-13(16),14-diene-2-one	*Hyattella* aff. *intestinalis*	Cytotoxicity against NBT-T2 cells, with an IC_50_ value of 24.1 µM	[47]
**1** **43**	6,10,18-Triacetoxy-2E,7E-dolabelladien	*Luffariella variabilis*	Cytotoxicity against the MDA-MB-231 cell line, with an IC_50_ value of 11.57 µM	[48]
**1** **45**	NN	*Pseudoaxinella flava*	Cytotoxicity against the PC3 cell line, with an IC_50_ value of 7 µM	[49]
**172**	3*β*-Hydroxyspongia-13(16),14-dien-2-one	*Spongia tubulifera*	Activity against A549, human skin melanoma A2058, hepatocyte carcinoma HepG2, and pancreas carcinoma MiaPaca-2 cell lines, with IC_50_ values of 88.1, 71.4, 91.3, and 90.0 µM, respectively	[50]
**1** **91**	Epoxynorspongian E	*Spongia* sp.	Activity against the PC3 and PBL-2H3 cell lines, with IC_50_ values of 24.8 and 27.2 µM, respectively	[51]
**199**	2*β*,3*α*,19-Triacetoxy-17-hydroxyspongia-13(16),14-diene	*Spongia officinalis* Linnaeus, 1759	Cytotoxicity against the K562 cell line, with an IC_50_ value of 7.3 µM	[52]
**206**	Ceylonamide G	*Spongia* sp.	Inhibited the growth of DU145 cells in two-dimensional monolayer culture, with an IC_50_ of 6.9 µM; also effective on spheroids of a three-dimensional DU145 cell culture model with a minimum effective concentration of 10 µM	[53]
**209**–**211**	Gracilins J–L	*Spongionella* sp.	Cytotoxic activity against K562 cells and normal human peripheral blood mononuclear cells (PBMCs), with IC_50_ values of 15 and 30, 8.5 and 9, and 2.65 and 3 µM, respectively	[54]
**212**	3′-Norspongiolactone	*Spongionella* sp.	Cytotoxic activity against K562 cells and normal PBMCs, with IC_50_ values of 12 and 30 µM, respectively	[54]
**2** **13**	Spongionellol A	*Spongionella* sp.	Activity in the cell lines PC3, PC3-DR, DU145, DU145-DR, 22Rv1, VCaP, and LNCaP, with IC_50_ values of 0.96, 1.23, 0.94, 1.53, 2.64, 1.30, and 1.02 µM, respectively	[55]
**2** **24**	Luakuliide A	Unidentified	Activity against HL-60 cells, with an IC_50_ value of 21.7 µM	[56]
**22** **7**	Chromodorolide D	Unidentified	Cytotoxicity against the NBT-T2 cell line, with an IC_50_ value of 5.6 µg/mL	[57]
**22** **8**	NN	Unidentified	Cytotoxicity against the NBT-T2 cell line, with an IC_50_ value of 12 µg/mL	[57]
			Antibacterial activity	
**25**	10-Hydro-9-hydroxyagelasine F	*Agelas nakamurai*	Inhibited the growth of *Mycobacterium smegmatis*, with inhibition zones of 10 mm at 20 µg/disc	[58]
**33**	(+)-10-Epiagelasine B	*Agelas citrina*	Active against *Staphylococcus aureus* ATCC 29213, *S*. *aureus* USA300LAC, *Streptococcus pneumoniae* ATCC 49619, *S*. *pneumoniae* 549 CHUAC, *Enterococcus faecalis* ATCC 29212, *E*. *faecalis* 256 CHUAC, and *E*. *faecium* 214 CHUAC, with MIC (minimum inhibitory concentration) values of 1, 2, 4, 8, 4, 4, and 4 µg/mL, respectively	[59]
**36**	(+)-Agelasine B	*Agelas mauritiana*	Active against a panel of methicillin-resistant *S. aureus* (MRSA) clinical strains 2010-260, 2010-210, 2010-292, and 2010-300, as well as a methicillin-susceptible *S. aureus* strain H608, with MIC_90_ values of 2, 1, 2, 1, and 2 µg/mL, respectively	[37]
**40**	Iso-agelasine C	*Agelas nakamurai*	Antibacterial activities against *Proteusbacillus vulgaris*, with an MIC value of 18.75 µg/mL	[38]
**46**–**49**	Agelasines O–R	*Agelas* sp.	Inhibited the growth of *S*. *aureus* and *Bacillus subtilis*, with MIC values of 16 and 16, 32 and 32, 8 and 8, and 8 and 8 µg/mL, respectively	[60]
**51**	Agelasine T	*Agelas* sp.	Inhibited the growth of *S*. *aureus* and *B*. *subtilis*, with MIC values of 16 and 16 µg/mL, respectively	[60]
**66**	Eleganstone A	*Dactylospongia elegans*	Antibacterial activity against *Escherichia coli*, *B. subtilis*, and *S. aureus*, with an MIC value of 64 µg/mL	[61]
**67**	(1R*,2*E*,4R*,8*E*,10S*, 11S*,12R*)-10,18-diacetoxydolabella-2,8-dien-6-one	*Dactylospongia elegans*	Antibacterial activity against *E. coli*, *B. subtilis*, and *S. aureus*, with an MIC value of 64 µg/mL	[61]
**8** **5**	Dendrillin B	*Dendrilla antarctica*	Achieved 90% eradication at 100 µg/mL in the MRSA biofilm assay	[62]
**88**	Darwinolide	*Dendrilla membranosa*	Cytotoxicity against MRSA, with an MIC of 132.9 μM, and activity against the biofilm formation of the same MRSA strain, with an IC_50_ value of 33.2 µM	[63]
**13** **8**	Monamphilectine A	*Hymeniacidon* sp.	Showed 43% and 38% of the bactericidal strength of the *β*-lactam antibiotics carbenicillin and amphicillin, respectively, against *E. coli* at a concentration of 150 nM	[64]
**203**	Spongenolactone A	*Spongia* sp.	Exhibited 46%, 47%, and 93% inhibition against *S. aureus* at 50, 100, and 200 µM, respectively	[65]
**204**	Spongenolactone B	*Spongia* sp.	Displayed 24%, 42%, and 40% inhibition against *S. aureus* at 50, 100, and 200 µM, respectively	[65]
			Antituberculosis activity	
**57**–**59**	Macfarlandins F–H	*Chelonaplysilla* sp.	Inhibited *Mycobacterium tuberculosis*, with MIC values of >20, 49, and >20 µg/mL, respectively	[66]
**13** **8**	Monamphilectine A	*Hymeniacidon* sp.	Activity against *M. tuberculosis* H_37_Rv, with an MIC value of 15.3 µg/mL	[64]
**21** **9**	7-Methylaminoisoneoamphilecta-1(14),15-diene	*Svenzea flava*	Activity against *M. tuberculosis* H_37_Rv, with an MIC value of 15 µg/mL	[67]
**2** **20**	7-Formamidoisoneoamphilecta-1(14),15-diene	*Svenzea flava*	Activity against *M. tuberculosis* H_37_Rv, with an MIC value of 32 µg/mL	[67]
			Antifungal activity	
**40**	Iso-agelasine C	*Agelas nakamurai*	Activity against *Candida albicans*, with an MIC value of 4.69 µg/mL	[38]
**41**	Iso-agelasidine B	*Agelas nakamurai*	Activity against *C*. *albicans*, with an MIC value of 2.34 µg/mL	[38]
**46**	Agelasine O	*Agelas* sp.	Inhibited the growth of *Trichophyton mentagrophytes* and *Cryptococcus neoformans*, with IC_50_ values of 32 and 16 µg/mL, respectively	[60]
**47**	Agelasine P	*Agelas* sp.	Inhibited the growth of *C*. *neoformans*, with an IC_50_ value of 32 µg/mL	[60]
**48**–**49**	Agelasines Q–R	*Agelas* sp.	Inhibited the growth of *Aspergillus niger*, *T*. *mentagrophytes*, *C*. *albicans*, and *C*. *neoformans*, both with IC_50_ values of 16, 16, 16, and 8 µg/mL, respectively	[60]
**51**	Agelasine T	*Agelas* sp.	Inhibited the growth of *C*. *neoformans*, with an IC_50_ value of 16 µg/mL	[60]
			Antiparasitic activity	
**60**	8-Isocyanoamphilecta-11(20),15-diene	*Ciocalapata* sp.	Activity against *Plasmodium falciparum* K1, with an IC_50_ value of 0.98 µM	[68]
**61**	(1*S*,3*S*,4*R*,7*S*,8*S*,11*S*,12*S*,13*S*,15*R*,20*R*)-7-Formamido-20-isocy-anoisocycloamphilectane	*Cymbastela hooperi*	Inhibitory effects on three strains of *P. falciparum* (FCR3F86, W2, and D6), with an average IC_50_ value of 0.5 µg/mL	[69]
**62**	(1*S*,3*S*,4*R*,7S,8*S*,11*S*,12*S*,13*S*,15*R*,20*R*)-7,20-Diformamidoisocy-cloamphilectane	*Cymbastela hooperi*	Inhibitory effects on *P. falciparum* FCR3F86, with an IC_50_ value of 14.8 µg/mL	[69]
**8** **3**	Membranoid B	*Dendrilla antarctica*	Activity against *Leishmania donovani* (IC_50_ 0.8 µM), with no discernible cytotoxicity against uninfected J774A.1 cells	[70]
**8** **4**	Membranoid D	*Dendrilla antarctica*	Activity against *L*. *donovani* (IC_50_ 1.4 µM), with no discernible cytotoxicity against uninfected J774A.1 cells	[70]
**8** **5**	Dendrillin B	*Dendrilla antarctica*	Activity against the leishmaniasis parasite, with an IC_50_ value of 3.5 µM	[62]
**92**	Diacarperoxide H	*Diacarnus megaspinorhabdosa*	Activity against *P*. *falciparum* (W2 clones) in vitro, with an IC_50_ value of 12.9 µM	[71]
**93**	Diacarperoxide I	*Diacarnus megaspinorhabdosa*	Activity against *P*. *falciparum* (W2 and D6 clones) in vitro, with IC_50_ values of 4.8 and 7.9 µM, respectively	[71]
**94**	Diacarperoxide J	*Diacarnus megaspinorhabdosa*	Activity against *P*. *falciparum* (W2 and D6 clones) in vitro, with IC_50_ values of 1.8 and 1.6 µM, respectively	[71]
**13** **8**	Monamphilectine A	*Hymeniacidon* sp.	Activity against a chloroquine-resistant (CQ-R) *P*. *falciparum* W2 strain, with an IC_50_ value of 0.60 µM	[64]
**217**–**218**	Monamphilectines B–C	*Svenzea flava*	Activity against *P. falciparum*, with IC_50_ values of 44.5 and 43.3 nM, respectively	[72]
			Anti-inflammatory activity	
**133**–**134**	Hipposponlachnins A–B	*Hippospongia lachne*	Inhibitory activity on the release of *β*-hexosaminidase in DNP-IgE-stimulated RBL-2H3 cells, with IC_50_ values of 49.37 and 23.91 µM, respectively	[73]
**141**	Erectcyanthin B	*Hyrtios erectus*	Inhibited 5-LOX, COX-2, and COX-1, with IC_50_ values of 0.88, 0.98, and 1.09 mM, respectively	[74]
**1** **93**	Sponalactone	*Spongia officinalis*	Inhibitory activity on the lipopolysaccharide (LPS)-induced nitric oxide (NO) production in RAW264.7 macrophages, with an IC_50_ value of 32 µM	[75]
**1** **94**	17-O-acetylepispongiatriol	*Spongia officinalis*	Inhibitory activity on the LPS-induced NO production in RAW264.7 macrophages, with an IC_50_ value of 15 µM	[75]
**1** **95**	17-O-acetylspongiatriol	*Spongia officinalis*	Inhibitory activity on the LPS-induced NO production in RAW264.7 macrophages, with an IC_50_ value of 12 µM	[75]
**1** **96**	15*α*,16*α*-Dimethoxy-15,16-dihydroepispongiatrio	*Spongia officinalis*	Inhibitory activity on the LPS-induced NO production in RAW264.7 macrophages, with an IC_50_ value of 22 µM	[75]
**19** **7**	15*α*-Ethoxyepispongiatriol-16(15*H*)-one	*Spongia officinalis*	Inhibitory activity on the LPS-induced NO production in RAW264.7 macrophages, with an IC_50_ value of 12 µM	[75]
**198**	17-Dehydroxysponalactone	*Spongia* sp.	Inhibited superoxide anion generation (91%) and elastase release (90%) at 10 µM, with IC_50_ values of 3.37 and 4.07 µM, respectively	[76]
**203**–**205**	Spongenolactones A–C	*Spongia* sp.	Inhibitory activity against superoxide anion generation in fMLF/CB-stimulated human neutrophils, with IC_50_ values of 16.5, 13.1, and 17.4 µM, respectively	[65]
**2** **21**	Tedanol	*Tedania ignis*	Showed anti-inflammatory activity through the inhibition of carrageenan-induced paw edema in mice	[77]
			Antifouling activity	
**11**–**16**	Kalihinols O–T	*Acanthella cavernosa*	Activity against *Balanus amphitrite* larvae, with EC_50_ (50% effective concentration) values of 1.43, 0.72, 1.48, 1.16, 0.53, and 0.74 µM, respectively	[32]
**42**	(−)-Agelasine D	*Agelas nakamurai*	Inhibited the growth of planktonic forms of the biofilm-forming bacteria *Staphylococcus epidermidis* (MIC < 0.0877 µM), but did not inhibit biofilm formation	[39]
**43**	(−)-Agelamide D	*Agelas nakamurai*	Inhibited only the biofilm formation but not the growth of *S. epidermidis*	[39]
**81**	9,11-Dihydrogracilin A	*Dendrilla antarctica*	Reduced the area covered by the fouling organisms	[78]
**82**	9,11-Dihydrogracillinone A	*Dendrilla antarctica*	Reduced the area covered by the fouling organisms	[78]
**13** **9**	Hymerhabdrin A	*Hymerhabdia* sp.	Toxic against larvae of the barnacle *Balanus amphitrite*, with an IC_50_ of 3.6 µg/mL	[79]
			Inhibition of osteoclasts	
**153**–**154**	Ceylonamides A–B	*Spongia ceylonensis*	Inhibitory effects on RANKL-induced osteoclastogenesis in RAW264 macrophages, with IC_50_ values of 13 and 18 µM, respectively	[80]
**1** **60**	Ceylonin A	*Spongia ceylonensis*	Inhibited the RANKL-induced formation of multinuclear osteoclasts in RAW264 cells by 70% (50 µM), in a dose-dependent manner, without cytotoxicity	[81]
**163**–**165**	Ceylonins D–F	*Spongia ceylonensis*	Inhibited the RANKL-induced formation of multinuclear osteoclasts by 28%, 47%, and 31%, respectively	[81]
			Others	
**43**	(−)-Agelamide D	*Agelas nakamurai*	Enhanced the radiation sensitivity of Hep3B cells; enhanced the efficacy of radiotherapy in a hepatocellular carcinoma (HCC) xenograft mouse model	[41]
**53**–**55**	Agelasines W–Y	*Astrosclera willeyana*	Inhibited the Cbl-b protein that negatively regulates T-cell activation, with IC_50_ values of 57, 72, and 66 µM, respectively	[82]
**1** **16**	Halioxepine	*Haliclona* sp.	Activity against 1,1-diphenyl-2-picrylhydrazyl (DPPH), with an IC_50_ of 3.2 µg/mL	[45]
**141**	Erectcyanthin B	*Hyrtios erectus*	Scavenged DPPH and ABTS^+^, with IC_50_ values of 0.45 and 0.40 mM, respectively	[74]
**141**	Erectcyanthin B	*Hyrtios erectus*	Activity against 3-hydroxy-3-methylglutaryl-coenzyme A reductase, with an IC_50_ value of 0.07 mM	[74]
**1** **44**	Niphateolide A	*Niphates olemda*	Inhibited the p53-Hdm2 (human Mdm2) interaction, with an IC_50_ value of 16 µM	[83]
**146**	Raspadiene	*Raspailia bouryesnaultae*	Inhibited the replication of HSV-1 (KOS and 29R strains) at 100 µg/mL by 83% and 74%, respectively	[84]
**1** **51**	18-Nor-3,5,17-trihydroxyspongia-3,13(16),14-trien-2-one	*Spongia* sp.	Inhibited aromatase in a dose-dependent manner, with an IC_50_ value of 34.4 µM	[85]
**1** **51**	18-Nor-3,5,17-trihydroxyspongia-3,13(16),14-trien-2-one	*Spongia* sp.	Doubled the quinone reductase 1 (QR1) activity in cultured Hepa 1c1c7 cells at 11.2 µM	[85]
**209**	Gracilin J	*Spongionella* sp.	Restored mitochondrial activity of neurons to control levels of 98.9% ± 4.7% (*p* < 0.001) at 0.1 µM, reversing the 28.6% ± 3.4% decrease caused by 200 µM H_2_O_2_ treatment	[86]
**2** **14**	26-O-etfhylstrongylophorine-14	*Strongylophora strongilata*	Inhibiting protein tyrosine phosphatase 1B (PTP1B), associated with type 2 diabetes, with an IC_50_ value of 8.7 µM	[87]

* Not named.

### 3.2. Acanthodendrilla

**Figure 4:**
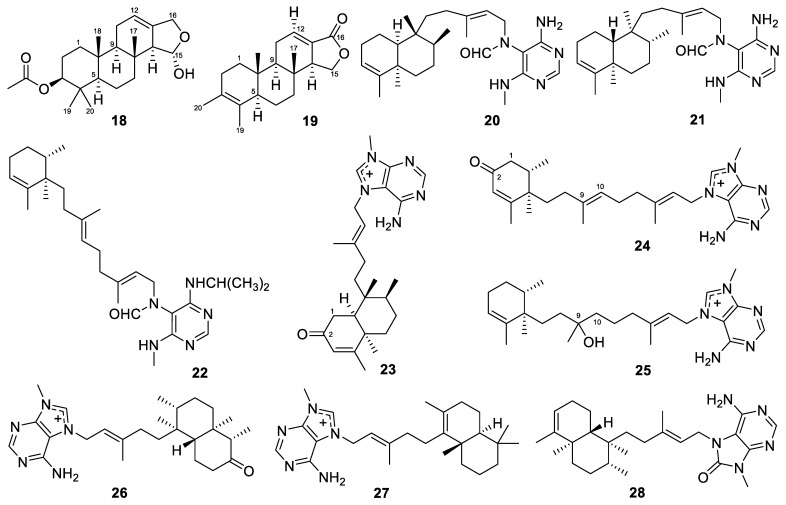
Chemical structures of diterpenes from *Acanthodendrilla* (**18**–**19**) and *Agelas* (**20**–**28**) sponges.

Only two diterpenes were reported to be generated by the *Acanthodendrilla* sponges. These two molecules are spongian diterpenes, named 3*β*-acetoxy-15-hydroxyspongia-12-en and 3-methylspongia-3,12-dien-16-one (**18**–**19**, Figure 4). They were isolated from the marine sponge *Acanthodendrilla* sp., collected in Pulau-Pulau [88]. Compounds **18** and **19** represent new chemical entities of the known spongian diterpene family. Compound **18** is a new 3-acetoxyspongia, while compound **19** represents an unreported rearranged 3-methylspongia-3-en. This is the first report of spongian diterpenes from the *Acanthodendrilla* genus. The cytotoxic potential of compounds **18** and **19** against A549, MDA-MB-231, human colorectal carcinoma cells (HT-29), and human pancreatic adenocarcinoma cells (PSN1) was evaluated. Both compounds proved to be inactive in the tested cancer cell lines. Their GC_50_ values (drug concentration causing a 50% reduction in the net protein increase) were over 27.5 and 33.3 µM, respectively, for all cell lines.

### 3.3. Agelas

*Agelas* sponges are prevalent in tropical and subtropical marine environments and are a significant source of bioactive natural products. The sponges of the genus *Agelas* have yielded a diverse array of metabolites, with 355 new ones reported between 1971 and 2021 [89]. Among over 19 species of *Agelas* sponges, *A. mauritiana* and *A. oroides* are the most prolific, yielding 45 and 36 metabolites respectively [90]. Specifically, regarding diterpenes, from 2009 to 2022, a total of 33 molecules were isolated from *Agelas* sponges, a number second only to those isolated from the genus *Spongia*.

Three pyrimidine diterpenes, named axistatins 1–3 (**20**–**22**, Figure 4), were isolated from the Palauan marine sponge *Agelas axifera* Hentschel [34]. All of the isolated compounds were found to be inhibitors of cancer cell growth. Axistatins 1–3 showed inhibition against the murine lymphocytic leukemia P388, pancreatic adenocarcinoma BXPC-3, breast adenocarcinoma MCF-7, CNS glioblastoma SF-268, lung large-cell carcinoma NCI-H460, colon adenocarcinoma KM20L2, and prostate carcinoma DU-145 cell lines, with GI_50_ values of 19.8, 22.8, and 8.9; 4.8, 5.5, and 6.0; 5.7, 6.8, and 5.8; 3.6, 3.9, and 3.5; 4.6, 4.3, and 5.4; 4.1, 4.1, and 6.9; and 4.8, 5.0, and 7.5 µM, respectively. Axistatins 1–3 also exhibited antimicrobial activity. A brief review has introduced promising anti-CNS-tumor active substances derived from marine sponges that were published between 1994 and 2014 [91]. Axistatins 1–3 are among the eight compounds featured in this review.

Three *N*-methyladenine-containing diterpenes, named 2-oxoagelasines A and F (**23**–**24**, Figure 4) and 10-hydro-9-hydroxyagelasine F (**25**), were isolated from the Okinawan marine sponge *Agelas nakamurai* Hoshino. Antibacterial experiments were performed on *Mycobacterium smegmatis* NBRC 3207 using the paper disc method. Compound **25** inhibited the growth of *M. smegmatis*, with an inhibition zone of 10 mm at 20 µg/disc [58].

Two *N*-methyladenine-containing diterpenes, nemoechines F–G (**26**–**27**, Figure 4), were isolated from the South China Sea sponge *Agelas* aff. *nemoechinata.* Compound **27** exhibited cytotoxicity against Jurkat cell lines, with an IC_50_ value of 17.1 µM [35].

Diterpenoid alkaloids are complex natural compounds that are primarily derived from specific plant genera and marine organisms, exhibiting a range of biological activities, from medicinal applications to potent neurotoxicity [92]. Marine sponges have also been reported to produce different diterpenoid alkaloids. From the South China Sea sponge *Agelas* aff. *nemoechinata*, a diterpene-adenine alkaloid called nemoechine D (**28**, Figure 4) was obtained [36]. It showed cytotoxicity against the HL-60 cell line, with an IC_50_ value of 9.9 µM.

**Figure 5:**
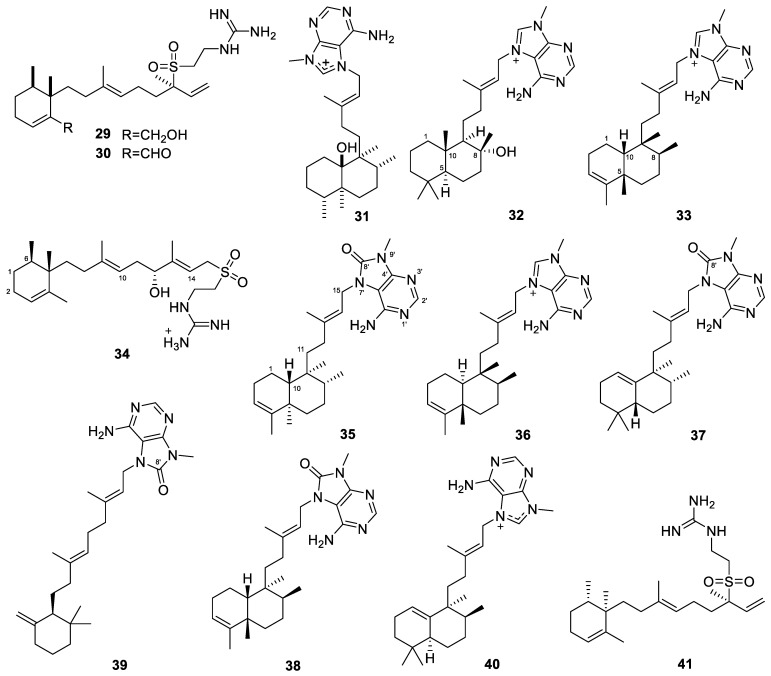
Chemical structures of diterpenes from *Agelas* sponges (**29**–**41**).

Three diterpene alkaloids, agelasidines E–F and agelasine N (**29**–**31**, Figure 5), were isolated from the Caribbean sponge *Agelas citrina* [93]. This represents the first report of natural products from the sponge *A. citrina*. Three diterpene alkaloids, (+)-8-epiagelasine T, (+)-10-epiagelasine B, and (+)-12-hydroxyagelasidine C (**32**–**34**, Figure 5), were also obtained from *A. citrina* [59]. The evaluation of antimicrobial activity against the Gram-positive pathogens *S. aureus*, *Streptococcus pneumoniae*, and *Enterococcus faecalis* showed that compound **33** was active against all of the tested strains, with MIC values in the range of 1–8 µg/mL.

Five diterpene alkaloids, (−)-8′-oxo-agelasine B, (+)-agelasine B, (+)-8′-oxo-agelasine C, agelasine V, and (+)-8′-oxo-agelasine E (**35**–**39**, Figure 5), were isolated from the sponge *Agelas mauritiana* [37]. Compounds **35** and **37**–**39** are the second examples of 8′-oxo-agelasine analogs. Compound **36** not only exhibited cytotoxicity toward the cancer cell lines PC9, A549, HepG2, MCF-7, and U937, with IC_50_ values of 4.49–14.07 µM, but also showed antibacterial activities against a panel of clinical MRSA isolates, with MIC values of 1–2 µg/mL (Table 1).

**Figure 6:**
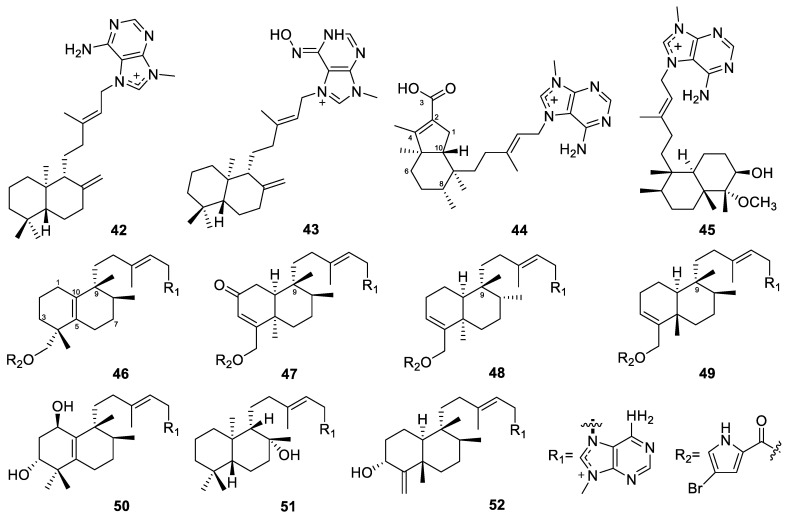
Chemical structures of diterpenes from *Agelas* sponges (**42**–**52**).

Two diterpene alkaloids, iso-agelasine C and iso-agelasidine B (**40**–**41**, Figure 5), were isolated from the South China Sea sponge *Agelas nakamurai* [38]. Compound **40** showed cytotoxicity against the HL-60, K562, and HCT-116 cell lines, with IC_50_ values of 25.3, 28.9, and 38.8 µM, respectively. Compound **41** showed cytotoxicity against the HL-60 and K562 cell lines, with IC_50_ values of 33.0 and 39.2 µM, respectively. Compound **40** also exhibited antibacterial activities against *Proteusbacillus vulgaris*, with an MIC value of 18.75 µg/mL. Moreover, compounds **40** and **41** showed antifungal activities against *Candida albicans*, with MIC values of 4.69 and 2.34 µg/mL, respectively. Another two diterpene alkaloids, (−)-agelasine D and (−)-ageloxime D (**42**–**43**, Figure 6), were also obtained from the *Agelas nakamurai* sponge [39]. They exhibited cytotoxicity against L5178Y mouse lymphoma cells, with IC_50_ values of 4.03 and 12.5 µM, respectively. Moreover, compound **42** inhibited the growth of planktonic forms of the biofilm-forming bacteria *Staphylococcus epidermidis* (MIC < 0.0877 µM), but it did not inhibit biofilm formation, whereas compound **43** showed the opposite activity profile and inhibited only biofilm formation but not bacterial growth.

It is worth mentioning that the reported compound **43,** named (−)-ageloxime D, was revised as (+)-*N*-[4-amino-6-(methylamino)pyrimidin-5-yl]-*N*-copalylformamide, which was produced via hydrolysis of agelasine D [40]. It was later renamed (−)-agelamide D [41]. (−)-Agelasine D was more cytotoxic to Hep3B cells than (−)-agelamide D, with their GI_50_ values being 9.9 and 12.0 µM, respectively [41]. It was found that (−)-agelamide D enhanced the radiation sensitivity of Hep3B cells, reducing their ability to form colonies and boosting the rate of apoptosis [41]. It also upregulated the expression of protein kinase RNA-like endoplasmic reticulum kinase/inositol-requiring enzyme 1*α*/activating transcription factor 4 (PERK/eIF2*α*/ATF4), a pivotal pathway in the unfolded protein response (UPR) across various HCC cell lines, thereby intensifying the UPR signaling triggered by radiation. In vivo xenograft studies validated that (−)-agelamide D amplified the suppressive effect of radiation on tumor growth, without causing systemic toxicity. Immunohistochemistry confirmed that (−)-agelamide D elevated ATF4 expression and the incidence of apoptosis induced by radiation, aligning with the in vitro observations.

Diterpene alkaloids were also found from an Okinawan marine sponge (*Agelas* sp.) [94]. Agelamasine A (**44**, Figure 6) is the first diterpene alkaloid with a rearranged (4→2)-*abeo*-clerodane skeleton from a marine source, while agelamasine B (**45**, Figure 6) is a clerodane diterpene alkaloid. Agelasines O–U (**46**–**52**, Figure 6) are also diterpene alkaloids isolated from the Okinawan marine sponge *Agelas* sp. [60]. Agelasines O–R were the third examples of diterpene alkaloids with a 9-*N*-methyladenine and a pyrrole unit. Agelasine O has a halimane skeleton, while agelasines P–R have a clerodane skeleton. Agelasines S–U are new diterpene alkaloids with a 9-*N*-methyladenine unit consisting of a halimane skeleton, a labdane skeleton, and a clerodane skeleton, respectively. Agelasines O–R and T showed antimicrobial activities against several bacterial and fungal strains (Table 1).

### 3.4. Astrosclera

**Figure 7:**
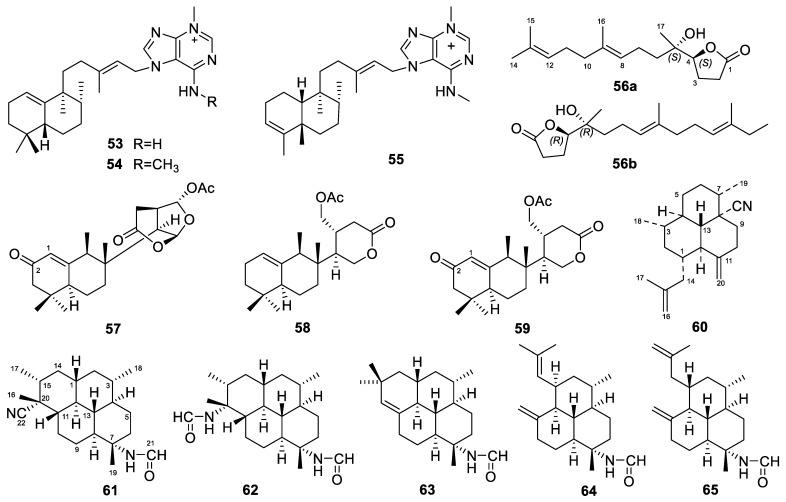
Chemical structures of diterpenes from *Astrosclera* (**53**–**55**), *Cacospongia* (**56**), *Chelonaplysilla* (**57**–**59**), *Ciocalapata* (**60**), and *Cymbastela* (**61**–**65**) sponges.

The *Astrosclera* sponges yielded three diterpenes in total. The three *N*-methyladenine-containing diterpenes, agelasines W–Y (**53**–**55**, Figure 7), were isolated from a specimen of *Astrosclera willeyana* collected in 1997 and frozen ever since. Agelasines W–Y have bicyclic terpenoid skeletons with a prenyl side chain that terminates with an *N*-methyladenine subunit. In terms of activity, these three compounds can inhibit the Cbl-b protein, with IC_50_ values of 57, 72, and 66 µM, respectively [82]. Cbl-b negatively regulates T-cell activation and, thus, lowers the immune system’s reaction to cancer cells. Agelasines W–Y could be promising immunotherapy agents for enhancing antitumor immunity by inhibiting the Cbl-b protein.

### 3.5. Cacospongia

Two unusual C_17_ *γ*-lactone norditerpenoids (a pair of inseparable enantiomers, **56a** and **56b**, Figure 7) were obtained from the marine sponge *Cacospongia* sp. as a mixture [95]. They bore the 4*S*,5*S* and 4*R*,5*R* absolute configurations, respectively. It should be noted that since these two compounds exist in the form of a mixture, they have been counted as one when tallying the number of diterpenes from different genera of sponges.

### 3.6. Chelonaplysilla

The *Chelonaplysilla* sponges yielded three diterpenes in total. These three diterpenes, macfarlandins F–H (**57**–**59**, Figure 7), were obtained from a sample of the marine sponge *Chelonaplysilla* sp. collected in Samoa [66]. Structurally, macfarlandins F and H are the first members of this family to have oxygenation at C-2, and macfarlandins G and H are the first to have a monocyclic *δ*-lactone heterocycle attached to their decalin ring system. In addition, macfarlandin G exhibited activity against *Mycobacterium tuberculosis*, with an MIC of 49 µg/mL, whereas macfarlandins F and H both exhibited MICs > 20 µg/mL.

### 3.7. Ciocalapata

Only one diterpene was reported to be produced by the *Ciocalapata* sponge. This molecule is a rare isonitrile diterpene named 8-isocyanoamphilecta-11(20),15-diene (**60**, Figure 7), which was isolated from a cryopreserved sample of a *Ciocalapata* sp. sponge [68]. The antimalarial activity of isonitrile terpenoids has long been reported. Coincidentally, compound **60** possesses strong activity against *Plasmodium falciparum* K1, with an IC_50_ value of 0.98 µM.

### 3.8. Cymbastela

The total number of diterpenes reported from the *Cymbastela* sponges is five. These five diterpene formamides (**61**–**65**, Figure 7) were obtained from the tropical marine sponge *Cymbastela hooperi* [69]. Compound **61** ((1*S*,3*S*,4*R*,7*S*,8*S*,11*S*,12*S*,13*S*,15*R*,20*R*)-7-formamido-20-isocyanoisocycloamphilectane) contains both formamide and isonitrile functionalities, which is not usual for natural products. Through in vitro antiplasmodial bioassays, compound **61** was found to have better activity (IC_50_ 0.5 µg/mL) than compound **62** ((1*S*,3*S*,4*R*,7*S*,8*S*,11*S*,12*S*,13*S*,15*R*,20*R*)-7,20-diformamidoisocycloamphilectane) (IC_50_ 14.8 µg/mL), whereas compounds **63**–**65** were inactive.

### 3.9. Dactylospongia

**Figure 8:**
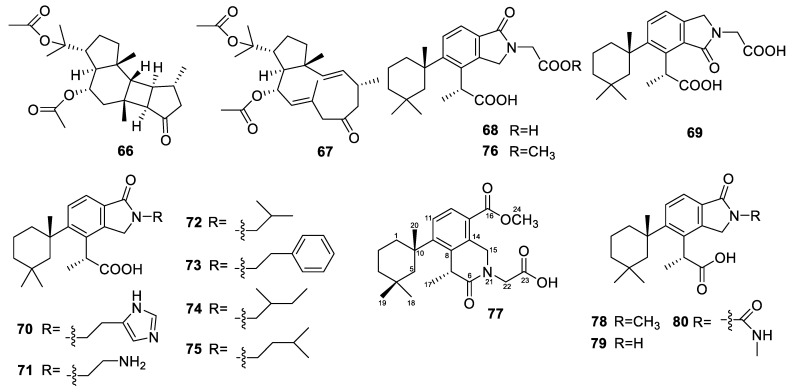
Chemical structures of diterpenes from *Dactylospongia elegans* (**66**, **67**) and *Darwinella* (**68**–**80**) sponges.

The *Dactylospongia* sponges yielded two diterpenes in total. These two diterpenes were isolated from the marine sponge *Dactylospongia elegans*. Eleganstone A (**66**, Figure 8) is a rare diterpene with a 5/6/4/5 fused tetracyclic ring skeleton, and compound **67** belongs to the dolabellane diterpenes, which had not been discovered from the genus *Dactylospongia* previously [61]. The antibacterial activity of these two compounds was evaluated, and their MIC values were 64 µg/mL against *E. coli*, *B. subtilis*, and *S. aureus*.

### 3.10. Darwinella

The *Darwinella* sponges yielded 13 diterpenes in total. Nine nitrogenous spongian diterpenes—oxeatamide A (**68**, Figure 8), iso-oxeatamide A (**69**), oxeatamides B-G (**70**–**75**), and oxeatamide A 23-methyl ester (**76**)—were isolated from the sponge *Darwinella oxeata* [96]. They were tested for cytotoxicity in a 48 h MTT (3-(4,5-dimethylthiazol-2-yl)-2,5-diphenyltetrazolium bromide) assay against HL-60 cells, and they all showed IC_50_ values >10 µM.

Four rearranged diterpenoids, oxeatine (**77**, Figure 8) and oxeatamides H–J (**78**–**80**), were isolated from the sponge *Darwinella* cf. *oxeata* [97]. Oxeatine has a new heterocyclic skeleton, and oxeatamide J has an *N*-methyl urea group included in a *γ*-lactam moiety. The 4,8-dimethyl-5-(1,3,3-trimethylcyclohexyl)-octahydro-1*H*-2*λ*^2^-isoquinoline heterocyclic skeleton found in oxeatine, which features a *δ*-lactam with the nitrogen atom bridging C-6 and C-15 of a rearranged spongian carbon framework, was unknown in nature or from synthesis.

### 3.11. Dendrilla

**Figure 9:**
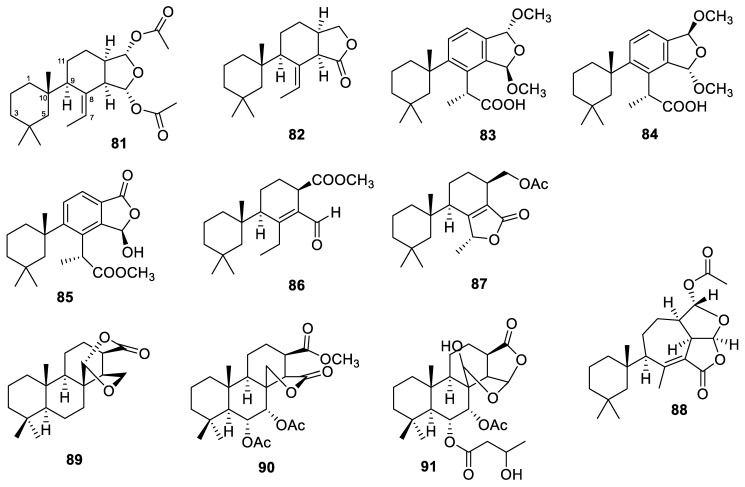
Chemical structures of diterpenes from *Dendrilla* sponges (**81**–**91**).

A total of 11 diterpenes were obtained from *Dendrilla* sponges. Two norditerpenes, named 9,11-dihydrogracilin A (**81**, Figure 9) and 9,11-dihydrogracillinone A (**82**), were isolated from the Antarctic sponge *Dendrilla antarctica* [78].

Their antifouling ability was tested using soluble-matrix paints, and both compounds showed activity against a variety of colonizing organisms. Compound **82** demonstrated a more pronounced antifouling effect, with a smaller area covered by the solitary colonial ascidian (*Botrylloides* sp.) compared to compound **81**.

Two diterpenes bearing one or more methyl acetal functionalities, membranoids B and D (**83**–**84**, Figure 9), were also obtained from the *Dendrilla antarctica* sponge [70]. They displayed low micromolar activity (IC_50_ values of 0.8 and 1.4 µM, respectively) against *Leishmani donovani*, with no discernible cytotoxicity against uninfected J774A.1 cells. Membranoids B and D are considered to be artifacts and can be obtained by treating aplysulfurin with methanol. Dendrillins B–D (**85**–**87**, Figure 9) were also produced by the *D. antarctica* sponge [62]. Dendrillin B showed an IC_50_ of 3.5 µM against the leishmaniasis parasite. Moreover, it achieved 90% eradication at 100 µg/mL in the MRSA biofilm assay.

A rearranged spongian diterpene, darwinolide (**88**, Figure 9), has been isolated from the Antarctic Dendroceratid sponge *Dendrilla membranosa* [63]. A broth dilution assay determined darwinolide’s MIC against MRSA to be 132.9 μM. It was also found that darwinolide was cytotoxic, rather than cytostatic, toward *S. aureus*. Further experiments revealed an IC_50_ value of 33.2 µM against the biofilm formation of the same MRSA strain. Hence, the compound darwinolide displays 4-fold selectivity against the biofilm phase of MRSA compared to the planktonic phase.

Three spongian diterpenes, named aplyroseols 20–22 (**89**–**91**, Figure 9), were isolated from two specimens of the Australian marine sponge *Dendrilla rosea* [98]. The 3-hydroxybutyrate present in aplyroseol 22 was reported in the spongian diterpenes for the first time. The compounds were screened for activity against *Staphylococcus aureus*, but they were inactive at 64 µg/mL.

### 3.12. Diacarnus

**Figure 10:**
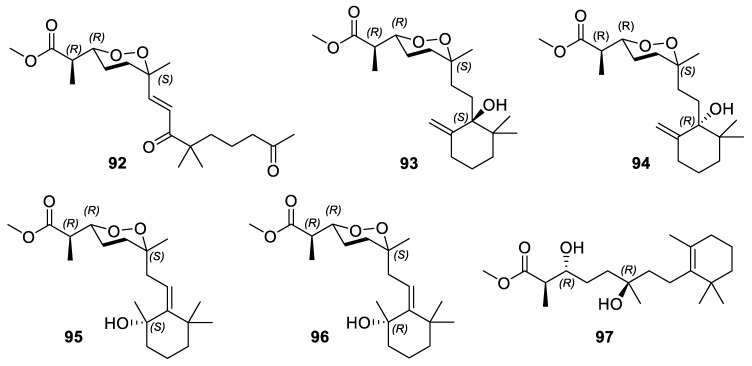
Chemical structures of diterpenes from the sponge *Diacarnus megaspinorhabdosa* (**92**–**97**).

The *Diacarnus* sponges yielded six diterpenes in total. These included five norditerpene endoperoxides, diacarperoxides H–L (**92**–**96**, Figure 10), together with a norditerpene diol, diacardiol B (**97**).

These were isolated from the South China Sea sponge *Diacarnus megaspinorhabdosa* [71]. Diacarperoxides H–J showed antimalarial activity against *Plasmodium falciparum* (W2 clones) in vitro, with IC_50_ values of 12.9, 4.8, and 1.8 µM, respectively, while diacarperoxides H–I exhibited activity against *P. falciparum* (D6 clones), with IC_50_ values of 7.9 and 1.6 µM, respectively. The IC_50_ values of the control drug artemisinin were 0.14 µM (W2 clones) and 0.071 (D6 clones).

### 3.13. Dysidea

**Figure 11:**
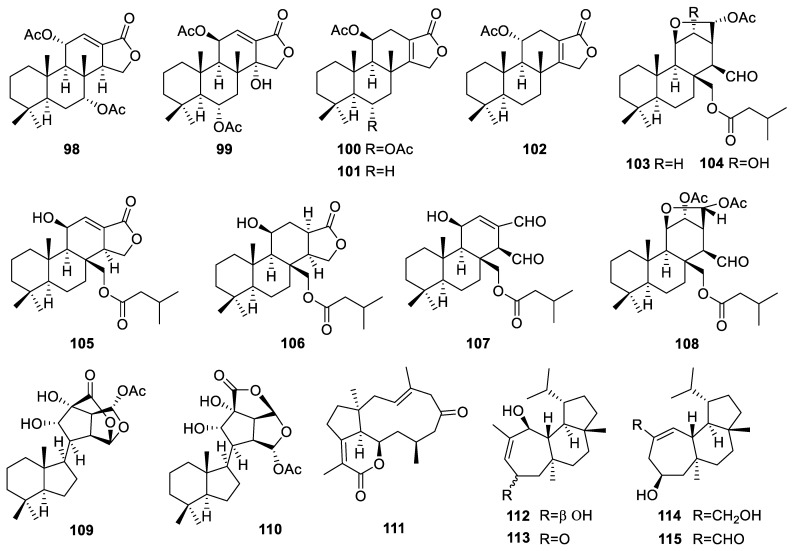
Chemical structures of diterpenes from *Dysidea* (**98**–**110**), *Fascaplysinopsis* (**111**), and *Halichondria* (**112**–**115**) sponges.

A total of 13 diterpenes were obtained from *Dysidea* sponges. Seven spongian-class diterpenes (**98**–**104**, Figure 11) were isolated from the sponge *Dysidea* cf. *arenaria* collected in Okinawa [42]. Compounds **99**, **103**, and **104** showed cytotoxicity against NBT-T2 rat bladder epithelial cells, with IC_50_ values of 1.9, 1.8, and 4.2 µg/mL, respectively. Four diterpenes, compounds **105**–**108** (Figure 11), were isolated from a sample of the *Dysidea* cf. *arenaria* sponge [43]. They were found to have inhibitory effects on NBT-T2 cells, with IC_50_ values of 3.1, 1.9, 8.4, and 3.1 µM, respectively. Chromodorolides D–E (**109**–**110**, Figure 11) were also generated by the *Dysidea* sp. sponge [99].

### 3.14. Fascaplysinopsis

Only one diterpene was reported to be produced by the *Fascaplysinopsis* sponge. The dolabellane diterpenoid, named clavirolide H (**111**, Figure 11), was isolated from the Xisha sponge *Fascaplysinopsis reticulata* [100]. Clavirolide H was evaluated for cytotoxic activities against human leukemia K562, HL-60, HeLa, HCT-116, A549, normal human hepatocytes L-02, and human hepatocellular carcinoma BEL-7402 cell lines, but no activity was observed.

### 3.15. Halichondria

A total of four diterpenes were obtained from *Halichondria* sponges. These four homoverrucosane-type diterpenes (**112**–**115**, Figure 11), common in land plants but rare in sponges, were isolated from a sample of the marine sponge *Halichondria* sp. [44]. This was the first report of homoverrucosanes isolated from the marine sponge *Halichondria* sp. Compounds **112**–**115** were evaluated for their activities against the RPMI-8266 cell line, and their IC_50_ values were all greater than 10 µM.

### 3.16. Haliclona

**Figure 12:**
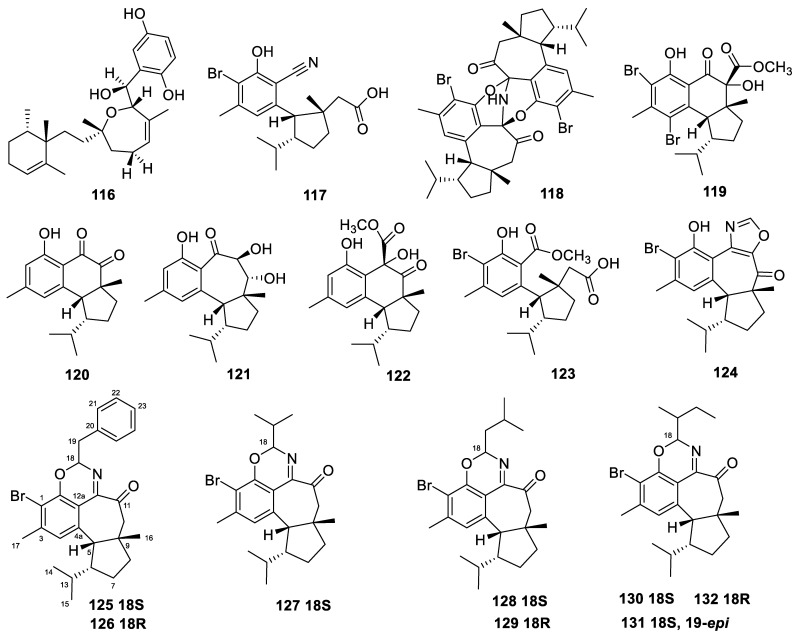
Chemical structures of diterpenes from *Haliclona* (**116**) and *Hamigera* (**117**–**132**) sponges.

Only one diterpene was reported from the *Haliclona* sponges. The marine sponge genus *Haliclona* contains over 600 species, but only a small number of them have been classified and chemically investigated [21]. The diterpenoid halioxepine (**116**, Figure 12) is a meroditerpene produced by *Haliclona* sp. [45]. It showed cytotoxicity against NBT-T2 cells (RIKEN), with an IC_50_ value of 4.8 µg/mL. It also showed antioxidant activity against DPPH, with an IC_50_ of 3.2 µg/mL.

### 3.17. Hamigera

The *Hamigera* sponges yielded 16 diterpenes in total. *Hamigera* sponges, belonging to the Hymedesmiidae family, are second only to the genus *Phorbas* in the same family in terms of the richness of obtained secondary metabolites [16].

The 16 hamigeran diterpenoids were isolated from the New Zealand marine sponge *Hamigera tarangaensis* (**117**–**132**, Figure 12) [46,101]. The compound hamigeran R (**117**) was the first benzonitrile-based marine natural product, while the compound hamigeran S (**118**) was the first dimeric structure in the series. The formation of hamigerans R and S is thought to occur via the reaction of hamigeran G with a nitrogen source, where the nitrile carbon of hamigeran R is derived from the terpenoid skeleton.

The compound hamigeran M (**124**) represents the first instance of a non-benzo-fused, oxazole-containing terpenoid isolated from the marine environment. Compounds **124**–**130** were tested for their cytotoxicity against the HL-60 cell line using the MTT method, and their IC_50_ values were 6.9, 19.5, 14.1, 14.7, 21.3, 11.6, and 33.3 µM, respectively.

### 3.18. Hippospongia

**Figure 13:**
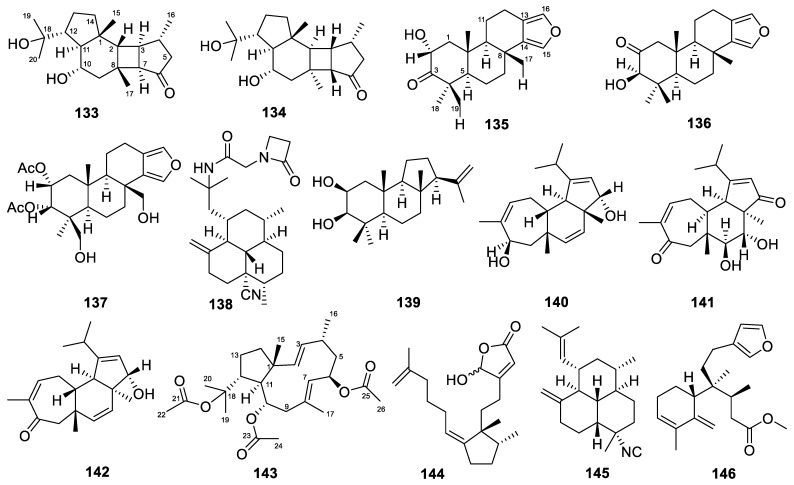
Chemical structures of diterpenes from *Hippospongia* (**133**–**134**), *Hyattella* (**135**–**137**), *Hymeniacidon* (**138**), *Hymerhabdia* (**139**), *Hyrtios* (**140**–**142**), *Luffariella* (**143**), *Niphates* (**144**), *Pseudoaxinella* (**145**), and *Raspailia* (**146**) sponges.

Two diterpenes were produced by *Hippospongia* sponges. These two diterpenes, hipposponlachnins A and B (**133**–**134**, Figure 13), with antiallergic activity, were obtained from the South China Sea marine sponge *Hippospongia lachne* [73]. They possess an unprecedented tetracyclo [9.3.0.0^2,8^.0^3,7^] tetradecane ring system. Hipposponlachnins A and B did not cause significant cytotoxicity in rat basophilic leukemia (RBL-2H3) cells after 24 h of treatment. They showed inhibitory activity on the release of *β*-hexosaminidase in DNP-IgE-stimulated RBL-2H3 cells, with IC_50_ values of 49.37 and 23.91 µM, respectively, higher than that of the market-available anti-asthmatic drug ketotifen fumarate (IC_50_ = 63.88 µM). In addition, hipposponlachnins A and B also suppressed IL-4 production in a dose-dependent manner and significantly inhibited LTB4 release in activated RBL-2H3 cells compared with untreated controls. The results indicate that these are promising antiallergic lead compounds.

### 3.19. Hyattella

The *Hyattella* sponges yielded three diterpenes in total. These three diterpenes, named 2*α*-hydroxyspongia-13(16),14-diene-3-one (**135**, Figure 13), 3*β*-hydroxyspongia-13(16),14-diene-2-one (**136**), and 2*α*,3*α*-diacetoxy-17,19-dihydroxyspongia-13(16),14-diene (**137**), were extracted from the sponge *Hyattella* aff. *intestinalis* [47]. They were tested against adenovirus (AdV), and all showed IC_50_ values > 20 µg/mL. Their cytotoxicity was also examined against NBT-T2 cells using the MTT assay, and compound **136** showed an IC_50_ value of 24.1 µM.

### 3.20. Hymeniacidon

Only one diterpene was produced by *Hymeniacidon* sponges. The molecule monamphilectine A (**138**, Figure 13) is a diterpenoid *β*-lactam alkaloid generated by *Hymeniacidon* sp. [64]. When tested against a CQ-R *Plasmodium falciparum* W2 strain, it showed an IC_50_ value of 0.60 µM. In vitro antituberculosis screening against *Mycobacterium tuberculosis* H_37_Rv revealed an MIC value of 15.3 µg/mL. Preliminary KB assays against *E. coli* revealed that, at a concentration of 150 nM, monamphilectine A possesses 43% and 38% of the bactericidal strength of the *β*-lactam antibiotics carbenicillin and amphicillin, respectively.

### 3.21. Hymerhabdia

Similarly, only one diterpene was reported from the *Hymerhabdia* sponges. The unusual diterpene hymerhabdrin A (**139**, Figure 13) was isolated from an intertidal marine sponge *Hymerhabdia* sp. [79]. It possessed a novel 6/6/5 fused-ring skeleton. Hymerhabdrin A exhibited antifouling activity against *Balanus amphitrite* larvae, with an LC_50_ (lethal concentration 50) value of 3.6 µg/mL.

### 3.22. Hyrtios

The *Hyrtios* sponges yielded three diterpenes in total. These three cyanthiwigin-type diterpenes, named erectcyanthins A–C (**140**–**142**, Figure 13), were isolated from the marine sponge *Hyrtios erectus* [74]. The compound erectcyanthin B not only exhibited anti-dyslipidemia activity but also possessed antioxidant and anti-inflammatory activities. In the anti-dyslipidemia experiment, erectcyanthin B exhibited activity against 3-hydroxy-3-methylglutaryl-coenzyme A reductase, with an IC_50_ value of 0.07 mM, compared to 0.08 mM for the drug atorvastatin. The antioxidant activities of erectcyanthin B, assessed using the stable DPPH and ABTS^+^ scavenging tests, were indicated by IC_50_ values of 0.45 and 0.40 mM, compared to 1.51 and 1.70 mM of the standard *α*-tocopherol, respectively. The anti-inflammatory activity of erectcyanthin B (IC_50_ 0.88–1.09 mM, Table 1), assessed using 5-LOX and isoforms of COX-2/1, was superior to that of other erectcyanthin analogs.

### 3.23. Luffariella

Only one diterpene was generated by *Luffariella* sponges. The dolabellane diterpene was obtained from the South China Sea sponge *Luffariella variabilis* and was named 6,10,18-triacetoxy-2*E*,7*E*-dolabelladien (**143**, Figure 13) [48]. This is the first dolabellane-type diterpenoid from the genus *Luffariella*. Compound **143** showed cytotoxicity against the MDA-MB-231 cell line, with an IC_50_ value of 11.57 µM.

### 3.24. Niphates

Similarly, only one diterpene was reported from the *Niphates* sponges. This compound, named niphateolide A (**144**, Figure 13), was isolated from the marine sponge *Niphates olemda* [83]. The inhibitory effect of compound **144** on the p53–Hdm2 interaction was examined using ELISA. It inhibited the interaction, with an IC_50_ value of 16 µM. Genetic mutations within the p53 tumor suppressor pathway are a common occurrence in human tumors. Mdm2/Hdm2 functions as an E3 ubiquitin ligase for p53 within the ubiquitin–proteasome system. The activity of niphateolide A in inhibiting the p53–Hdm2 interaction enables it to serve as a substance for reactivating p53, thereby giving it potential for further research and development in the field of oncology.

### 3.25. Pseudoaxinella

*Pseudoaxinella* is another genus of sponge that has produced only one diterpene. The isonitrile diterpene (**145**, Figure 13), with anticancer activity, was isolated from the Caribbean sponge *Pseudoaxinella flava* [49]. Compound **145** showed growth-inhibitory activity against human PC3 prostate cancer cells, with an IC_50_ value of 7 µM.

### 3.26. Raspailia

*Raspailia* sponges also produced one diterpene in total. The clerodane diterpene, named raspadiene (**146**, Figure 13), was isolated from the marine sponge *Raspailia bouryesnaultae* collected in South Brazil [84]. The evaluation of potential anti-herpes activity against herpes simplex virus type 1 (HSV-1) showed that the compound raspadiene at 100 µg/mL inhibited the replication of HSV-1 (KOS and 29R strains, sensitive and resistant to acyclovir, respectively) by 83% and 74%, respectively.

### 3.27. Spongia

The *Spongia* genus produced the highest number of diterpenoids among all sponge genera. A total of 62 diterpenoids have been derived from *Spongia* sponges, accounting for 27% of all sponge-derived diterpenoids reported from 2009 to 2022. Many of these molecules exhibit strong biological activity, highlighting the potential and significance of *Spongia* sponges as a valuable resource for marine drug development.

**Figure 14:**
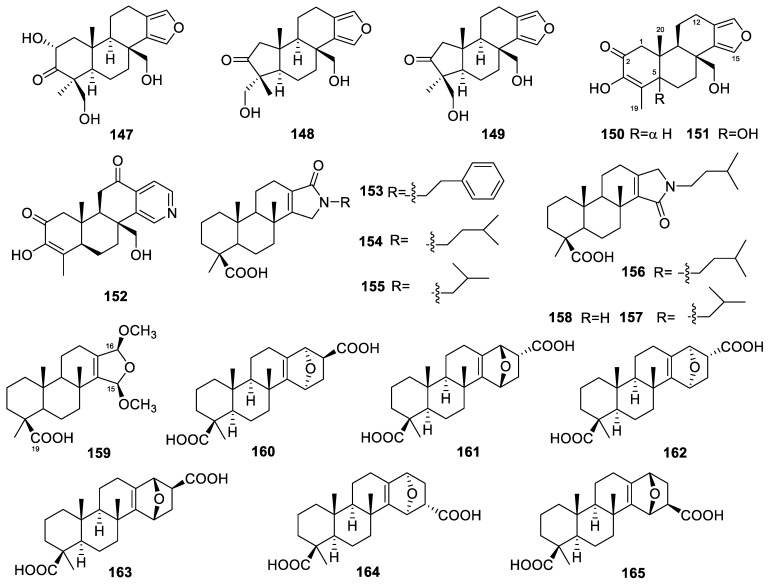
Chemical structures of diterpenes from *Spongia* sponges (**147**–**165**).

Three unreported furanoditerpenoids (**147**–**149**, Figure 14) were isolated from the marine sponge *Spongia* sp. [102]. Compound **147** is a spongian diterpene with a modified oxidation pattern, while compounds **148** and **149** represent two new ring-A-contracted spongians, displaying a novel and unprecedented norspongian carbon skeleton.

Three compounds, 18-nor-3,17-dihydroxyspongia-3,13(16),14-trien-2-one (**150**, Figure 14), 18-nor-3,5,17-trihydroxyspongia-3,13(16),14-trien-2-one (**151**), and spongiapyridine (**152**), were isolated from an Indonesian sponge of the genus *Spongia* [85]. Structurally, the D-ring possessed by compound **152** belongs to the pyridyl ring system, which is different from ordinary spongians possessing standard *δ*-lactone. In the in vitro biological activity experiment, compound **151** exhibited aromatase-inhibitory activity, with an IC_50_ value of 34.4 µM, and it also induced quinone reductase 1 (QR1) activity in cultured Hepa 1c1c7 cells, with a CD value (the concentration needed to double the QR1 activity) of 11.2 µM. Aromatase is an essential cytochrome P450 enzyme that facilitates the conversion of androgens like testosterone and androstenedione into estrogens such as estradiol and estrone. Inhibiting aromatase reduces the available estrogen and demonstrates considerable effectiveness in preventing certain types of breast cancer. Quinone reductase 1 is a protective enzyme that helps prevent cancer by blocking intracellular semiquinone radicals and producing *α*-tocopherolhydroquinone, which acts as a chemopreventive agent. Compound **151** holds promise as a potential therapeutic agent for both breast cancer prevention and chemoprevention, due to its dual action as an aromatase inhibitor and an inducer of quinone reductase 1 activity.

**Figure 15:**
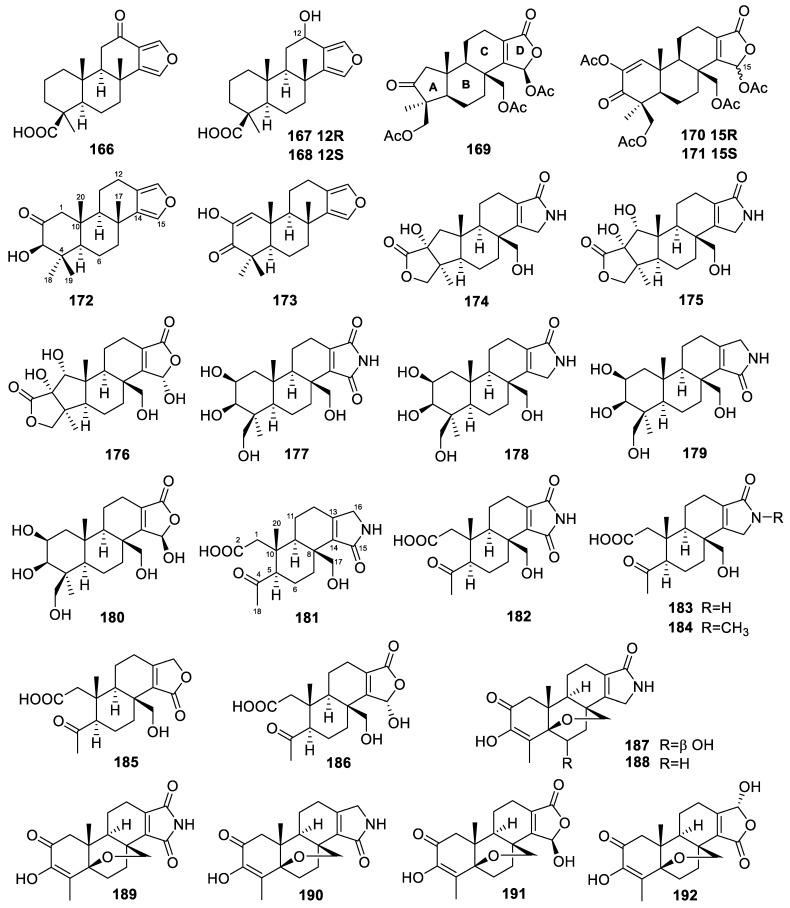
Chemical structures of diterpenes from *Spongia* sponges (**166**–**192**).

Seven spongian diterpenes, ceylonamides A–F (**153**–**158**, Figure 14) and 15*α*,16-dimethoxyspongi-13-en-19-oic acid (**159**), were isolated from the Indonesian marine sponge *Spongia ceylonensis* [80]. Compounds **153**–**158** are nitrogenous spongian diterpenes. Ceylonamides A and B exhibited inhibitory effects on RANKL-induced osteoclastogenesis in RAW264 macrophages, with IC_50_ values of 13 and 18 µM, respectively. In a follow-up study of the structure–activity relationship, the authors found that the carbonyl position of the *γ*-lactam ring and the volume of the substituent on the nitrogen atom had a great influence on the inhibitory effect.

Another nine spongian diterpene derivatives, ceylonins A–F (**160**–**165**, Figure 14) [81,103] and ceylonins G–I (**166**–**168**, Figure 15) [81,103], were also isolated from the *Spongia ceylonensis* sponge. Ceylonins A–F contain three additional carbons in ring D to supply an ether-bridged bicyclic ring system. Ceylonins A and D–F (50 µM) inhibited the RANKL-induced formation of multinuclear osteoclasts in RAW264 cells by 70%, 28%, 47%, and 31%, respectively. The inhibitory activity of ceylonins G–I against the cancer therapeutic drug target ubiquitin-specific protease 7 (USP7) was tested, but their IC_50_ values were all over 50 µM.

3-Nor-spongiolide A (**169**, Figure 15), having the rare 3-nor-spongian carbon skeleton, and spongiolides A and B (**170**–**171**), possessing a *γ*-butenolide ring instead of the usual furan ring for ring D, were isolated from South China Sea sponge *Spongia officinalis* [104]. They were evaluated for their cytotoxic activity against HL-60 cells, but none of them exhibited potent cytotoxic activity.

Two furanoditerpenes, 3*β*-hydroxyspongia-13(16),14-dien-2-one (**172**, Figure 15) and 19-dehydroxy-spongian diterpene 17 (**173**), were isolated from the sponge *Spongia tubulifera*, collected in the Mexican Caribbean [50]. Compound **172** showed activity against the A549, A2058, HepG2, and MiaPaca-2 cell lines, with IC_50_ values of 88.1, 71.4, 91.3, and 90.0 µM, respectively.

Spongiains A–G (**174**–**180**, Figure 15) were isolated from the marine sponge *Spongia* sp. [105]. Spongiains A–C were the first examples of spongian diterpenes bearing a pentacyclic skeleton composed of a fused 5/5/6/6/5 ring system through ring-A rearrangement. The cytotoxic activities of spongiains A–G were evaluated, and none of them exhibited antiproliferative effects on several cancer cell lines.

Twelve norspongian diterpenes, dinorspongians A–F (**181**–**186**, Figure 15) and epoxynorspongians A-F (**187**–**192**), were isolated from the marine sponge *Spongia* sp. [51]. Among them, dinorspongians A–F were the first examples of dinorspongian diterpenes bearing the unprecedented 3,4-seco-3,19-dinorspongian diterpene skeleton. Epoxynorspongian E exhibited inhibitory activity against the PC3 and PBL-2H3 cell lines, with IC_50_ values of 24.8 and 27.2 µM, respectively.

**Figure 16:**
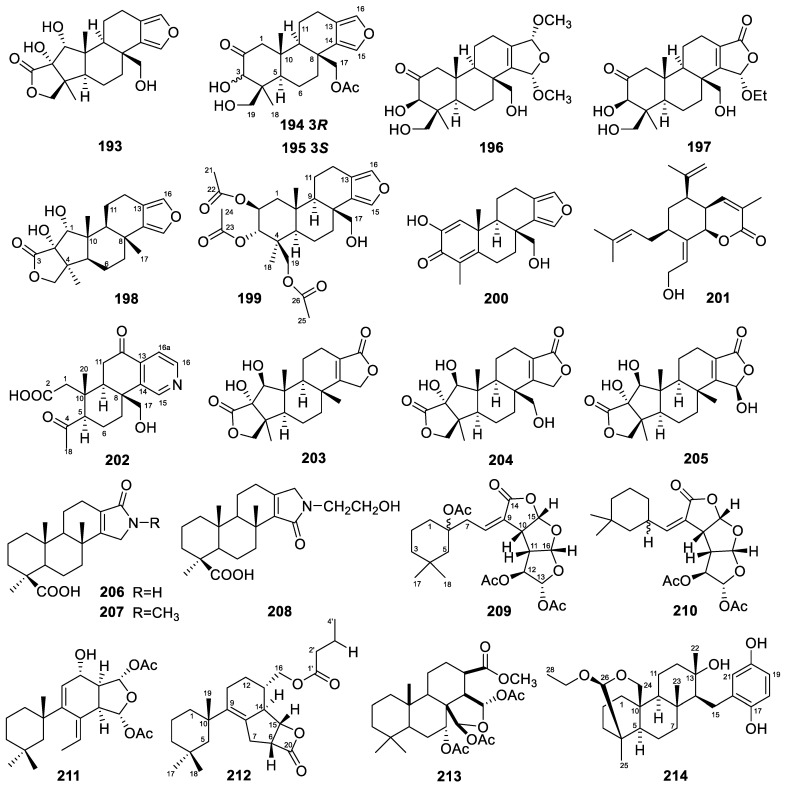
Chemical structures of diterpenes from *Spongia* (**193**–**208**), *Spongionella* (**209**–**213**), and *Strongylophora* (**214**) sponges.

Five diterpenes—including a 5,5,6,6,5-pentacyclic diterpene, sponalactone (**193**, Figure 16); two spongian diterpenes, 17-*O*-acetylepispongiatriol (**194**) and 17-*O*-acetylspongiatriol (**195**); and two spongian diterpene artifacts, 15*α*,16*α*-dimethoxy-15,16-dihydroepispongiatriol (**196**) and 15*α*-ethoxyepispongiatriol-16(15*H*)-one (**197**)—were isolated from the marine sponge *Spongia officinalis* collected from the South China Sea [75]. The in vitro anti-inflammatory activities of these compounds were tested by inhibition of LPS-induced NO production in RAW264.7 macrophages, with IC_50_ values of 32, 15, 12, 22, and 12 µM, respectively.

A rare A-ring contracted diterpene, 17-dehydroxysponalactone (**198**, Figure 16), was isolated from the Red Sea marine sponge *Spongia* sp. [76]. The in vitro anti-inflammatory activity of the compound was tested. Compound **198** significantly reduced the superoxide anion generation and elastase release, with inhibition rates of 91% and 90%, respectively, at a concentration of 10 µM, and the IC_50_ values were 3.37 and 4.07 µM, respectively.

A novel acetoxy diterpenoid, 2*β*,3*α*,19-triacetoxy-17-hydroxyspongia-13(16),14-diene (**199**, Figure 16), and 18-nor-2,17-hydroxyspongia-1,4,13(16),14-quaien-3-one (**200**), belonging to the rare 18-norspongian carbon skeleton, were isolated from the aquaculture *Spongia officinalis* Linnaeus, 1759 [52]. The in vitro biological activity evaluation showed that compound **199** had cytotoxic activity against the K562 cell line, with an IC_50_ value of 7.3 µM. A bicyclic diterpene, jellynolide A (**201**), with a penta-substituted carbon skeleton, was also isolated from this sponge [106]. The compound jellynolide A biogenically implies an irregular non-head-to-tail linkage between GPP (geranyl diphosphate) and isoprene units, as well as a novel cyclization position.

Dinorspongiapyridine (**202**, Figure 16) is a dinorspongian diterpene produced by the marine sponge *Spongia* sp. [107]. It was the first instance of a 3,4-seco-3,19-dinorspongian diterpene bearing a rare pyridyl D-ring system.

Spongenolactones A–C (**203**–**205**, Figure 16) were obtained from a Red Sea sponge *Spongia* sp. [65]. They are all pentacyclic spongian diterpenes, featuring 5,5,6,6,5, 5,5,6,6,6, and 5,5,6,6,7 ring systems, respectively. They were found to exert inhibitory activity against superoxide anion generation in fMLF/CB-stimulated human neutrophils, with IC_50_ values of 16.5, 13.1, and 17.4 µM, respectively. Furthermore, spongenolactone A showed higher inhibitory activity against the growth of *S. aureus* in comparison to B (Table 1).

Ceylonamides G–I (**206**–**208**, Figure 16) are diterpene alkaloids from an Indonesian marine sponge of *Spongia* sp. [53]. Ceylonamide G inhibited the growth of DU145 human prostate cancer cells in a two-dimensional monolayer culture, with an IC_50_ of 6.9 µM. It was also effective (minimum effective concentration of 10 µM) on spheroids of a three-dimensional cell culture model, which was prepared from DU145 cells.

### 3.28. Spongionella

The *Spongionella* sponges yielded five diterpenes in total. Four diterpenes, gracilins J–L and 3′-norspongiolactone (**209**–**212**, Figure 16), were isolated from the extracts of the marine *Spongionella* sp. sponges [54]. Gracilins J–L belong to the rare classes trisnorditerpenes, bisnorditerpenes, and norditerpenes, respectively. The in vitro cytotoxicity of these compounds was determined using K562 and normal human peripheral blood mononuclear cells (PBMCs). Compounds **209**–**212** showed cytotoxic activity against the K562 cell line, with IC_50_ values ranging from 2.65 to 15 µM (Table 1). They showed similar or slightly less toxicity against the normal PBMCs, with IC_50_ values ranging from 3 to 30 µM (Table 1). The activity of gracilins J–L was also evaluated using an oxidative in vitro stress model [86]. The compound gracilin J presented neuroprotection effects at the mitochondrial function level. The neurons’ mitochondrial activity decreased by 28.6 ± 3.4% (*p* < 0.001) after treatment with 200 µM H_2_O_2_. Gracilin J at 0.1 µM reduced this effect, restoring the activity to control levels (98.9 ± 4.7%, *p* < 0.001).

A spongian diterpene, spongionellol A (**213**, Figure 16), was obtained from the marine sponge *Spongionella* sp. [55]. It exhibited activity and selectivity in a panel of seven human prostate cancer cells. The panel included AR-negative PC3 and DU145 cells, which are known to be resistant to various hormonal and standard chemotherapeutics, docetaxel-resistant PC3-DR and DU145-DR cells (derived from PC3 cells and DU145 cells, respectively), AR-FL- (androgen receptor full-length) and AR-V7-positive (androgen receptor splice variant V7) hormone-resistant 22Rv1 and VCaP cells, and AR-FL-positive hormone-sensitive LNCaP cells (Table 1).

### 3.29. Strongylophora

Only one diterpene was reported to be produced by the *Strongylophora* sponge. This meroditerpene, 26-O-ethylstrongylophorine-14 (**214**, Figure 16), was isolated from the Caribbean marine sponge *Strongylophora strongilata* [87]. It was found to inhibit PTP1B associated with type 2 diabetes, with an IC_50_ value of 8.7 µM, compared with 0.7 µM for the positive control oleanolic acid. This is the first report of meroditerpenes inhibiting PTP1B activity.

### 3.30. Stylissa

**Figure 17:**
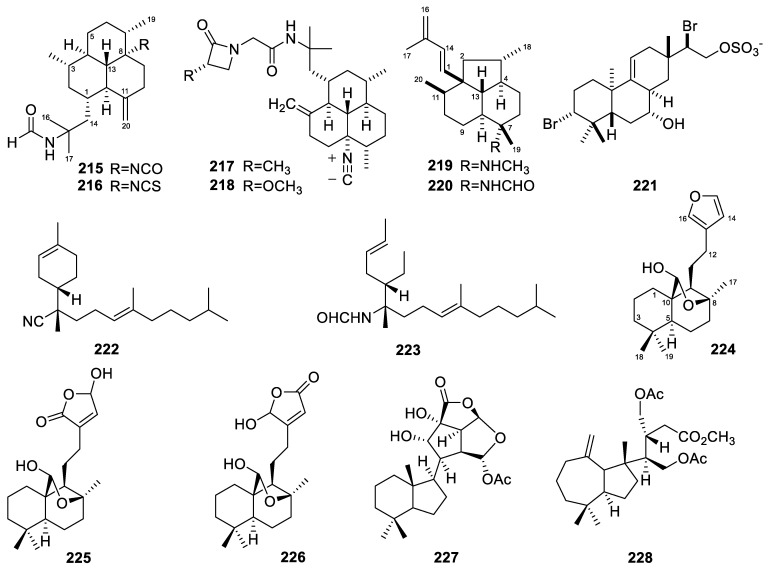
Chemical structures of diterpenes from *Stylissa* (**215**–**216**), *Svenzea* (**217**–**220**), *Tedania* (**221**), *Theonella* (**222**–**223**), and unidentified (**224**–**228**) sponges.

The *Stylissa* sponges yielded two diterpenes in total. These two amphilectane-type diterpenes, 8-isocyanato-15-formamidoamphilect-11(20)-ene and 8-isothiocyanato-15-formamidoamphilect-11(20)-ene (**215**–**216**, Figure 17), were isolated from the sponge *Stylissa* cf. *massa* [108]. Their antimalarial activities were evaluated, and both compounds were inactive.

### 3.31. Svenzea

A total of four diterpenes were obtained from *Svenzea* sponges. Two isocyanide amphilectane-type diterpenes, named monamphilectines B and C (**217**–**218**, Figure 17), were isolated from the Caribbean sponge *Svenzea flava* [72]. Monamphilectines B and C exhibited activities against the human malaria parasite *Plasmodium falciparum* (the non-resistant (wild-type standard) 3D7 strain), with IC_50_ values of 44.5 and 43.3 nM, respectively.

Two rare isoneoamphilectane-based diterpenes, 7-methylaminoisoneoamphilecta-1(14),15-diene and 7-formamidoisoneoamphilecta-1(14),15-diene (**219**–**220**, Figure 17), were also isolated from the Caribbean marine sponge *Svenzea flava* [67]. Their MIC values against the strain *Mycobacterium tuberculosis* H_37_Rv were 15 and 32 µg/mL, respectively.

### 3.32. Tedania

Only one diterpene was reported from *Tedania* sponges. The molecule, tedanol (**221**, Figure 17), was a brominated and sulfated pimarane diterpene isolated from the Caribbean sponge *Tedania ignis* [77]. Tedanol, which showed good solubility in water, significantly reduced both the acute (4 h) and subchronic (48 h) phases of carrageenan-induced paw edema in mice, which was coupled with a strong inhibition of COX-2 expression, cellular infiltration measured as myeloperoxidase (MPO) levels, and iNOS expression.

### 3.33. Theonella

The *Theonella* sponges yielded two diterpenes in total. The two nitrogenous prenylbisabolane diterpenes, named amitorines A and B (**222**–**223**, Figure 17), were isolated from *Theonella swinhoei* [109]. No activity data have been reported for them.

### 3.34. Others

A total of five diterpenes were isolated from other sponges, specifically those whose genera have not been determined.

The Kingdom of Tonga is an archipelago in the central Indo-Pacific Ocean; many novel marine natural products with bioactivities have been reported from organisms collected within Tongan territorial waters [110]. Coincidentally, three labdane diterpenes, luakuliides A–C (**224**–**226**, Figure 17), were isolated from a Tongan dictyoceratid marine sponge [56]. They share a *trans*-fused [4.4.0]-bicyclodecane system with a hemi-acetal functional group bridging C-8 and C-10. In terms of activity, luakuliide A has inhibitory activity against HL-60 cells, with an IC_50_ value of 21.7 µM.

Two diterpenes (**227**–**228**, Figure 17) were isolated from an Okinawan marine sponge [57]. Compound **227**, named chromodorolide D, is an example of a diterpenoid with a highly rearranged chromodorane carbon skeleton, while compound **228** retains the open side chains. Compounds **227** and **228** showed cytotoxicity against NBT-T2 cells, with IC_50_ values of 5.6 and 12 µg/mL, respectively.

## 4. Conclusions

This review summarizes the structures and bioactivities of 228 diterpenes reported in 73 research papers, originating from more than 33 different genera of sponges. Notably, the genera *Spongia*, *Agelas*, and *Acanthella* reported the highest number of diterpenes, with counts of 62, 33, and 17, respectively, accounting for 27%, 14%, and 7% of the total reported molecules, respectively. In contrast, in more than 26 genera of sponges, fewer than 10 diterpenoids have been reported per genus, with 12 of these genera producing just one diterpene each.

Most of the reported diterpenes have been evaluated for their bioactivity, with 110 molecules exhibiting one or more types of bioactivity. Among them, the highest numbers were seen in those with cytotoxic, antibacterial, and anti-inflammatory properties, amounting to 54, 22, and 13 molecules, respectively. These correspond to 24%, 10%, and 6% of the reported 228 molecules, respectively.

Some compounds exhibit cytotoxic activity against a variety of tumor cells. For example, the compounds axistatins 1–3 (**20**–**22**) have shown inhibitory activity against seven different tumor cell lines. Axistatins 1–2 have GI_50_ values ≤ 5 µM against five and four cell lines, respectively. Axistatin 3 has GI_50_ values ranging from 3.5 to 8.9 µM against seven cell lines. It is worth noting that, in addition to cytotoxic activity against tumor cells, the potential of some diterpenes as antitumor active molecules is reflected in their inhibitory activity against specific target proteins like Cbl-b, aromatase, etc. The production of cytotoxic metabolites by sponges may be beneficial for their self-protection, such as against predators and spatial competition [28].

Reports have documented the antibacterial, antifungal, antiviral, and antiparasitic activities of diterpenoids derived from various sources, such as sponges, fungi, and plants [111]. In our review of the literature on sponge-derived diterpenoids, we noticed that some molecules exhibit multiple activities. For instance, the compound (−)-agelamide D (**43**) exhibited cytotoxic activity against L5178Y mouse lymphoma cells and, in another activity evaluation model, demonstrated cytotoxic activity against Hep3B cells. It has also been proven to enhance the radiosensitivity of Hep3B cells. Furthermore, (−)-agelamide D also inhibits the biofilm formation of *S. epidermidis*. The compound iso-agelasine C (**40**) showed cytotoxicity against the HL-60, K562, and HCT-116 cell lines, exhibited antibacterial activity against *Proteusbacillus vulgaris*, and demonstrated antifungal activity against *Candida albicans*.

Some compounds have very low active concentrations. For example, monamphilectines B–C (**217**–**218**) exhibited antiparasitic activity against *P. falciparum*, with IC_50_ values of 44.5 and 43.3 nM, respectively. Compound (−)-agelasine D (**42**) inhibited the growth of planktonic forms of the biofilm-forming bacterium *Staphylococcus epidermidis*, with an MIC < 0.0877 µM. The compound spongionellol A (**213**) showed cytotoxicity against seven cancer cell lines, with IC_50_ values ranging from 0.94 to 2.64 µM. Kalihinols O–T (**11**–**16**) showed antifouling activity against *Balanus amphitrite* larvae, with EC_50_ values ranging from 0.53 to 1.48 µM.

The diversity in chemical structure and biological activity exhibited by these sponge-derived diterpenoids demonstrates their great potential in the development of marine drugs. However, the variety of activity evaluation models, while providing opportunities to discover different activities of these molecules, makes it challenging to compare the activities among different diterpenoids, and the structure–activity relationships are difficult to define. Activity concentration units such as µM, mM, and µg/mL further complicate the comparison of different studies. Moreover, there is no unified standard for defining the presence and strength of activity. Balancing standardization with diversity is a challenge. Additionally, most activity evaluations only characterize inhibition rates or IC_50_ values; further research to identify active targets and elucidate mechanisms of action will better facilitate the application and development of these molecules.
